# Imposing Phase II and Phase III Clinical Trials of Targeted Drugs for Glioblastoma: Current Status and Progress

**DOI:** 10.3389/fonc.2021.719623

**Published:** 2021-09-09

**Authors:** Yaning Wang, Wanqi Chen, Yixin Shi, Chengrui Yan, Ziren Kong, Yuekun Wang, Yu Wang, Wenbin Ma

**Affiliations:** ^1^Department of Neurosurgery, Peking Union Medical College Hospital, Chinese Academy of Medical Sciences and Peking Union Medical College, Beijing, China; ^2^Department of Neurosurgery, Peking University International Hospital, Beijing, China; ^3^Department of Head and Neck Surgery, National Cancer Center/National Clinical Research Center for Cancer/Cancer Hospital, Chinese Academy of Medical Sciences and Peking Union Medical College, Beijing, China

**Keywords:** glioblastoma, targeted therapy, phase III trial, phase II trial, overall survival

## Abstract

The most common primary intracranial tumor is glioma, among which glioblastoma (GBM) has the worst prognosis. Because of the high degree of malignancy of GBM and frequent recurrence after surgery, postoperative therapy, including chemotherapy, radiotherapy, targeted therapy, and immunotherapy, is particularly important. A wide variety of targeted drugs have undergone phase III clinical trials for patients with GBM, but these drugs do not work for all patients, and few patients in these trials have prolonged overall survival. In this review, some imposing phase III clinical trials of targeted drugs for glioma are introduced, and some prospective phase II clinical trials that have been completed or are in progress are summarized. In addition, the mechanisms of these drugs are briefly introduced, and deficiencies of these clinical trials are analyzed. This review aims to provide a comprehensive overview of current research on targeted drugs for glioma to clarify future research directions.

## Introduction

Glioma is the most common primary intracranial tumor, and the most common glioma histology is glioblastoma (GBM), which causes significant mortality and morbidity. The incidence of GBM is estimated to be approximately 3.21 individuals per 100,000 people (1). According to population-based studies, even with various treatment modalities including surgical resection, concurrent chemoradiotherapy and adjuvant chemotherapy, GBM patients have the poorest overall survival, with only 0.05% to 4.7% of patients surviving 5 years past diagnosis. Although there are standard treatment protocols, almost all patients experience tumor recurrence, and the average time to recurrence and progression is approximately 6 to 11 months (2, 3). Currently, targeted drugs are popular and widely studied in GBM, especially for recurrent GBM (rGBM). This review will briefly introduce some of these targeted drugs and their mechanisms in the treatment of glioma.

Targeted drugs refer to drugs that specifically target carcinogenic sites at the cellular or molecular level, bind to these targets and act on them. These targets may be molecules that play a key role in the occurrence and development of tumors and regulate signal transduction pathways. Drugs that target these molecules can specifically kill tumor cells without affecting normal tissue cells around the tumor, thus treating the tumor. Compared with chemotherapy, targeted drugs are advantageous because they can be selected based on the characteristics of different patients and do not target normal proliferative cells, but they are less broadly applicable than chemotherapy.

## Targeted Drugs With Completed Phase III Trials

### Vascular Endothelial Growth Factor Inhibitor

Many targeted drugs have already completed phase III clinical trials, among which the most widely used is bevacizumab (BEV). BEV is a monoclonal antibody that targets VEGF and is used as a monotherapy or in combination with a secondary agent in GBM (4). The US Food and Drug Administration (FDA) approved the use of BEV for rGBM in 2009 based on evidence from two phase II clinical trials, however, it was not approved by the European Medicines Agency (EMA) considering no sufficient evidence on survival benefit from BEV. Additionally, a phase III study of BEV, European Organization for the Research and Treatment of Cancer (EORTC) 26101, investigated the efficacy of BEV in patients with progressive GBM and showed that combining BEV with lomustine (LOM) did not confer an overall survival (OS) advantage over LOM alone but prolonged median progression-free survival (PFS) to some extent (5). In the combination group, patients received LOM at a dose of 90 mg per square meter of body surface area every 6 weeks. If there were no obvious hematologic toxic effects during the first cycle, the dose of LOM was increased to 110 mg for the second cycle, equal to the monotherapy group.

This outcome may be attributable to several factors, including the dosing difference in the first cycle, different treatments after disease progression and the detailed assessment of biomarkers in the patient group. IDH mutation status may also increase the sensitivity to treatment. However, most trials on rGBM have not analyzed patients according to IDH mutational status.

The addition of BEV to standard therapy for nGBM was investigated in the RTOG-0825 study (6). The study showed a 3.4-month extension of PFS, while first-line use of BEV did not improve OS. Moreover, declines in neurocognitive function and quality of life were observed in the BEV-treated group compared to the placebo group. Another phase III trial named AVAGlio (7) also showed that the addition of BEV to a standard regimen did not improve OS in patients with GBM. However, improved PFS and maintenance of baseline quality of life and performance status were observed with BEV. This is because a pseudo-response may occur in the early stage of treatment and such a “response” will also be present on MRI. At this point, if clinicians use MRI to assess the efficacy of BEV, the disease progression cannot be recognized (8).Thus, the phase III evidence for BEV is still disappointing.

Based on 52 relevant studies of BEV, a meta-analysis conducted by Diaz RJ et al. (9) found that although BEV could prolong PFS and OS in recurrent GBM patients either alone or in combination with cytotoxic agents, the survival advantage was limited to 4 months and no significant difference of survival was observed in primary setting. The authors also concluded that BEV might additionally benefit some patients from steroid sparing effects, however, BEV would also lead to several significant adverse effects and lose of potential practice of other therapeutic choices.

Cediranib, an oral pan-vascular endothelial growth factor receptor (VEGFR) tyrosine kinase inhibitor, was also investigated in a randomized, phase III trial either as monotherapy or in combination with lomustine versus lomustine in rGBM patients (10). Patients were randomly assigned in a 2:2:1 ratio to receive cediranib (30 mg) monotherapy, cediranib (20mg) in combination with lomustine (110mg/m2), or lomustine (110 mg/m2) in combination with placebo. The primary end point of PFS was not significantly different among these three groups, but this outcome may have been confounded by the use of post-progression BEV therapy. A total of 136 patients received post-progression anticancer therapy, with the majority receiving BEV either as monotherapy or in combination. BEV utilization was similar between the cediranib plus lomustine and placebo plus lomustine arms, but was less frequent in the cediranib monotherapy arm. Furthremore, the failure of this trial may also contribute to the proper use of the drug itself, which we will discuss in the following section.

### Other Molecular Targeted Drugs

Disappointing results were also obtained in the phase III trial for the platelet-derived growth factor receptor (PDGFR) inhibitor Imatinib. In a randomized phase 3 trial involving patients with progressive, rGBM for whom front-line therapy had failed, the primary study end point was not met, and no superior antitumor activity was noted for the combination therapy compared with HU monotherapy alone in the treatment of rGBM (11).

Enzastaurin is a macrocyclic bisindolylmaleimide that can target both PKC and PI3K/AKT pathways to suppress proliferation and tumor-induced angiogenesis (12, 13). An international phase III study involving 266 patients aimed to compare the efficacy and safety of enzastaurin versus LOM in rGBM patients. The study indicated that enzastaurin did not have superior efficacy to LOM in patients with rGBM (14).

Cilengitide is the main selective αvβ3 and αvβ5 integrin inhibitor that has been developed (15). Although the results from phase II studies showed a PFS as well as an OS benefit, negative outcomes were observed in the CORE trial for GBM patients with unmethylated MGMT promoters and in the CENTRIC trial involving 3471 patients with nGBM, indicating that the doses of cilengitide used in these studies were ineffective when combined with TMZ/RT-TMZ for GBM patients (16–18).

The mammalian target of rapamycin (mTOR) inhibitor everolimus can restrict cell growth and proliferation and proved effective in reducing the volume of subependymal giant cell astrocytomas in a phase III trial (19). However, several studies evaluating mTOR inhibition in GBM have reported disappointing results in regard to OS (20).

Epidermal growth factor receptor (EGFR) is associated with oncogenesis in glioblastoma, and substantial evidence supports a key role for EGFR in glioma progression. Approximately 60% of glioblastoma patients have some kind of genomic alteration affecting this pathway; of particular interest is the vIII mutation (21). Among small tyrosine kinase inhibitors (TKIs), Gefitinib, Afatinib and Lapatinib have shown very limited efficacy in rGBM (22–24) because of the brain penetrance and the lack of specificity, as TKIs often act on several tyrosine kinases, albeit with differential efficacy. Several antibodies developed against EGFR have been used against GBM. Nimotuzumab was the only one that was developed into a phase III trial. A multicenter, randomized phase III trial was conducted by Westphal M et al. to test the efficacy of additional nimotuzumab to standard RT/TMZ for nGBM (25). The study failed to reach the expected outcomes in PFS and OS, though it showed a trend toward efficacy in subgroups of unmethylated MGMT promoter patients. A purely immunological approach to target EGFRvIII was attempted by vaccination therapy (26). Unfortunately, the pivotal phase III trial for nGBM failed to show overall efficacy and was discontinued after interim analysis showed no significant benefit of the vaccine for OS (27).

Completed phase III evidence for targeted drugs used as monotherapy or combined with RT/TMZ, which is summarized in **Table 1**, does not suggest an appreciable survival benefit. The reasons of failure are analyzed and summarized in the following section. Thereafter, a literature review on prospective phase II clinical trials of targeted drugs in GBM is presented that highlights some notable drugs and clinical trials to provide a comprehensive understanding of the current status of targeted molecular therapies in newly diagnosed and recurrent GBM patients (**Table 2**). The characteristics of the included clinical trials are as follows: 1) the study populations are adult GBM patients; 2) the main objects of study are targeted drugs or drugs previously thought to be targeted; 3) the studies are a phase II clinical trials; 4) the results show prolonged OS or PFS in patients, including those screened for specific molecular markers, with acceptable side effects.

**Table 1:** Characteristics of imposing phase III clinical crials for GBM.

First author/Published year	Patients status (number)	Intervention (number)	Comparison (number)	Results	Study design
Wolfgang Wick et al., (5)	Recurrent GBM (437)	LOM + BEV (288)	LOM (149)	LOM + BEV: OS: 9.1m, PFS: 4.2m; LOM: OS: 8.6m, PFS: 1.5m; PFS was prolonged by adding BEV to LOM, but this study didn’t confer a survival advantage.	Phase III, randomized (EORTC 26101)
Mark R. Gilbert et al., (6)	Newly diagnosed GBM (637)	BEV+RT with TMZ, then TMZ (320)	Placebo+RT with TMZ, then TMZ (317)	BEV+RT with TMZ, then TMZ: OS: 15.7m, PFS: 10.7m; Placebo+RT with TMZ, then TMZ: OS: 16.1m, PFS: 7.3m; MGMT promoter status was a prognostic factor regardless of treatment.	Phase III, randomized (RTOG 0825)
Thomas Sandmann et al., (7)	Newly diagnosed GBM (349)	BEV+ RT + TMZ (171)	Placebo +RT + TMZ (178)	For patients with GBM, the proneural IDH1 wild-type molecular subtype can be benefit from BEV.	Phase III, randomized (Avaglio)
Tracy T. Batchelor et al., (10)	Recurrent GBM (325)	Cediranib(130); Cediranib + LOM(130)	LOM+Placebo (65)	The primary end point of PFS was not significantly different for either cediranib alone or cediranib in combination with lomustine versus lomustine.	Phase III, randomized (REGAL)
Wolfgang Wick et al., (14)	Recurrent GBM (266)	Enzastaurin (174)	LOM (92)	Median OS, PFS and 6-month PFS rate did not differ significantly between enzastaurin and LOM	Phase III, randomized
Gregor Dresemann et al., (11)	Recurrent GBM(240)	Imatinib + HU (120)	HU (120)	The median PFS for the combination arm was low at 6 weeks and similar to the monotherapy arm (6 weeks).	Phase III, randomized
Roger Stupp et al., (17)	Newly diagnosed GBM with methylated MGMT promoter (545)	Cilengitide+ RT + TMZ (272)	RT + TMZ (273)	Adding cilengitide to TMZ chemoradiotherapy cannot improve outcomes of newly diagnosed GBM patients with methylated MGMT promoter	Phase III, randomized
Manfred Westphal et al., (25)	Newly diagnosed GBM (142)	Nimotuzumab+ RT+ TMZ (71)	RT + TMZ (71)	Nimotuzumab + RT+ TMZ: OS: 22.3m, PFS: 7.7m;RT + TMZ: OS: 19.6m, PFS: 5.8m; There was a higher PFS rate in the experimental arm in the EGFR amplified cohort.	Phase III, randomized
Malkki, H. et al., (27)	Newly diagnosed GBM (745)	Rindopepimut +TMZ (371)	Placebo+ TMZ (374)	Rindopepimut +TMZ: OS: 20.4m;TMZ+Placebo: OS: 21.1m;The trial was discontinued after interim analysis showed no significant benefit of the vaccine on OS.	Phase III, randomized (ACT IV)

GBM, glioblastoma; RT, radiotherapy; TMZ, temozolomide; LOM, lomustine; Hydroxyurea, HU; OS: overall survival; PFS, progression free survival; MGMT, O6-Methylguanine-DNA Methyltransferase; Ona, onartuzumab; EGFR, epidermal growth factor receptor; IDH, isocitrate dehydrogenase; EORTC, European Organization for the Research and Treatment of Cancer; RTOG, the Radiation Therapy Oncology Group; REGAL, Recentin in Glioblastoma Alone and With Lomustine; REGAL, Recentin in Glioblastoma Alone and With Lomustine; m, month(s).

**Table 2:** Characteristics of imposing phase II clinical trials for GBM.

First author/Published year	Patients status (number)	Intervention (number)	Comparison (number)	Results	Study design
Giuseppe Lombardi et al., (28)	Recurrent GBM (119)	RT + TMZ + Regorafenib (59)	RT + TMZ + LOM (60)	Regorafenib: OS: 7.4m, 12-month overall survival rate: 38.9%;LOM: OS: 5.6m, 12-month overall survival rate: 15.0%;	Phase II, randomized (REGOMA)
Jaishri O. Blakeley et al., (29)	Newly diagnosed GBM (81)	RT + TMZ + Iniparib (81)	Historical control(the EORTC/NCIC phase III trial)^17,40,41^	The mOS was 21.6m, 2-year and 3-year survival rates are 38.3% and 24.7%;OS: MGMT promoter methylated: 30m; MGMT promoter unmethylated: 15.8m; MGMT promoter methylated status unknown: 25.9m.	Phase II, single arm
Xiao-Tang Kong et al., (30)	Newly diagnosed GBM (24)	RT + TMZ + Bortezomib (24)	Historical control^2,6,48^	mPFS: 6.2m; 6-month PFS rate: 54.2%;mOS: 19.1m (MGMT promoter methylated: 61m; MGMT promoter unmethylated: 16.4m)	Phase II, single arm
Thomas Kaley et al., (31)	Glioma patients with BRAFV600E mutation, including high grade glioma (24)	Vemurafenib (24)	Historical control	For the GBM and anaplastic astrocytoma cohort, the PFS and OS were 5.3m and 11.9m.Vemurafenib has clinical meaningful activity in glioma patients with BRAFV600E mutation.	Phase II, multi-cohort
Timothy Cloughesy et al., (32)	First recurrent, after chemoradiotherapy, bevacizumab-naïve patients with GBM (129)	Ona + BEV (64)	Placebo + BEV (65)	Ona + BEV: PFS: 3.9m, OS: 8.8m;Placebo + BEV: PFS: 2.9m, OS: 12.6m;In unselected patients with recurrent GBM, there was no evidence of further clinical benefit of Ona + BEV compared with BEV plus placebo.	Phase II, randomized
Wolfgang Wick et al., (33)	GBM patients at first or second progression (91)	rRT + APG101/CAN008 (58)	rRT (26)	rRT + APG101/CAN008: 6m PFS rate: 20.7%;rRT: 6m PFS rate: 3.8%;Methylation levels at CpG2 in the CD95L promoter in the tumor was concerned as a potential biomarker.	Phase II, randomized
Isabel Arrillage Romany (34)	Recurrent GBM, BEV-naïve, IDH1/2 wild type (17)	ONC monotherapy (17)	Historical meta-analyses^77-79^	Median OS was 41.6 weeks, 6m OS was 71%, 9m OS was 53%, 6m PFS was 11.8%.	Phase II, single arm
Reena P. Thomas (35)	Newly diagnosed GBM (29)	RT + TMZ + Plerixafor (29)	Historical control^2^	Median OS was 21.3m, median PFS was 14.5m. The drug was well-tolerated and no serious adverse effect was found in this trial.	Phase I/II, single arm

GBM, glioblastoma; RT, radiotherapy; rRT, reradiotherapy; TMZ, temozolomide; LOM, lomustine; OS, overall survival; mOS, median overall survival; PFS, progression free survival; mPFS, median progression free survival; MGMT, O6-Methylguanine-DNA Methyltransferase; Ona, onartuzumab; EGFR, epidermal growth factor receptor; m, month(s).

## Targeted Drugs With Promising Phase II Trials

### Multi-Kinase Inhibitor

As an oral multitarget tyrosine kinase inhibitor, regorafenib inhibits angiogenesis and other tumor-driven pathways, such as vascular endothelial growth factor receptor (VEGFR) 1-3, PDGFR, fibroblast growth factor receptor (FGFR), the angiopoietin receptor TIE-2, tyrosine kinase receptors, receptor tyrosine kinase genes, leukemia factor 1, the BRAF gene and other protein kinase activities (36, 37). Therefore, this drug can block tumor angiogenesis, inhibit tumor cell proliferation and control the tumor microenvironment, thus inhibiting tumor proliferation and invasion. Regorafenib has been approved by the FDA and the European Drug Administration (EMA) for metastatic colorectal cancer, gastrointestinal stromal tumors and primary hepatocellular carcinoma (38–40). In a number of previous clinical trials, researchers found that regorafenib had an antiglioma effect, which was related to the inhibition of angiogenesis and the PDGFR pathway (36). Regorafenib also underwent a phase I clinical trial with cetuximab for patients with advanced cancer, including a GBM patient who ultimately benefited from this regimen (41).

Based on the characteristics of this drug and the results of preclinical trials, Giuseppe Lombardi et al. conducted a randomized multicenter phase II clinical trial (28). This study included 119 patients with rGBM between November 2015 and February 2017 and randomly divided the 119 patients into two groups: one treated with regorafenib and the other was treated with LOM. OS was significantly improved in the regorafenib group (7.4 months *vs*. 5.6 months), but patients treated with regorafenib had more grade 3-4 adverse events (56% *vs*. 40%) than those treated with LOM. This drug is currently being evaluated in the up-front and recurrent setting in the ongoing GBM-AGILE phase III trial and is one of the options for treating rGBM patients under NCCN guidelines (Version 1.2020).

While the data from this trial appears promising, there are still some drawbacks include adverse effects (AEs). In the experimental group treated with regorafenib, grade 3-4 drug-related AEs were present in up to 56% of patients, which may be a potential concern for future large-scale clinical trials.

### Poly ADP-Ribose Polymerase Inhibitor in Disguise

Iniparib (4-iodo-3-nitrobenzamide) is a prodrug with anticancer activity that is mediated by mismatch repair defects. This drug was initially used for triple-negative breast cancer and breast cancer 2 (BRCA-2) mutant pancreatic cancer (42, 43), and recent studies have shown that this drug may have anti-tumor effects in other types of cancers, such as brain tumors. Iniparib was first used as a PARP inhibitor, and it was initially thought that the anti-tumor effect was produced solely by inhibiting PARP. However, later studies have shown that iniparib does not work by inhibiting PARP directly (44, 45). The anti-tumor effect is achieved by releasing an activated nitro radical ion that binds to selenium proteins, including thioredoxin reductase, which are key enzymes in redox reactions. This process leads to selective cytotoxicity. Therefore, even though iniparib may be a disguised PARP inhibitor, there is no denying that it has a clear anti-tumor effect on GBM.

Based on this antitumor mechanism, a multicenter single-arm phase II clinical trial involving 81 newly diagnosed GBM patients was conducted (29). In addition to concurrent chemoradiotherapy and adjuvant chemotherapy (TMZ), the regimen included iniparib (an initial dose of 6.8 mg/kg intravenously (i.v.) was given twice weekly, and a maintenance dose of 8.6 mg/kg i.v. was given twice weekly after determining its tolerability). The median OS (mOS) of the patients in this study was 22 months, and the 2-year and 3-year survival rates were 38.3% and 24.7%, respectively. The results indicated that a weekly dose of 17.2 mg/kg iniparib was tolerable in combination with radiotherapy and TMZ. Compared with the historical OS data obtained by EORTC/NCIC phase III trials (17, 46, 47), OS was significantly improved in this study, suggesting that iniparib has potential antitumor effects.

However, the conclusion is still controversial, as this trial was a single-arm trial. Randomization is important in designing clinical trials, and ensuring the unicity of variables between the experimental group and the control group is a prerequisite for accurate conclusions. Clearly, a single-arm trial does not meet these requirements. This clinical trial selected patients from the classic EORTC/NCIC phase III clinical trials as historical controls, but these clinical trials were completed several years ago, and with the development of various supportive therapies in recent years, OS prolongation has not been identified as a drug effect. Secondly, the calculation of OS varies among different clinical trials. Some clinical trials calculate OS from the time of diagnosis, while others calculate OS from the date of enrollment, which will also have an impact on the conclusion. Therefore, the reliability of the trial’s conclusion still needs to be further verified.

### Proteasome Inhibitors

To ensure that cells can proliferate normally, proteins in the cell that are abnormal often need to be degraded in a certain way. The ubiquitin proteasome pathway (UPP) is an important intracellular proteolytic pathway. The degradation of intracellular proteins by UPP accounts for approximately 80% of all intracellular protein degradation, and the hydrolysis process consists of two parts: protein ubiquitination and proteolysis (48). The 26S proteasome plays a major role in the hydrolysis process. In the process of tumorigenesis and development, the rapid proliferation of cells tends to increase the possibility of the aberrant protein expression. Therefore, the proteasome is especially important in tumor cells (49). The results of the present study are consistent with this conclusion. Proteasome levels and activities were much higher in primary tumor cells than those in normal tissues.

Based on this principle, proteasome inhibitors were developed. The first-generation proteasome inhibitor bortezomib inhibits the degradation of many proteins, resulting in disruption of the cell cycle. In addition, the misfolded proteins cannot be cleared by the proteasome in a timely manner, resulting in the disruption of many signal transduction pathways in cells. Therefore, bortezomib affects the cell cycle and apoptosis (50, 51). Moreover, bortezomib also inhibits cell adhesion, angiogenesis and cytokine-mediated intercellular communication, thus affecting the microenvironment during tumorigenesis. Bortezomib also affects chemotherapy, increasing the sensitivity of tumors to chemotherapy by inhibiting the NF-κB pathway and overcoming the problem of drug resistance to a certain extent in GBM according to some preclinical studies (52).

Xiao Tang et al. conducted a phase II clinical trial on bortezomib combined with radiotherapy for the treatment of newly diagnosed GBM (30). A total of 24 patients were enrolled, and all patients received standard concurrent chemoradiotherapy, followed by 24 cycles of TMZ until their tumors progressed. During radiotherapy, bortezomib was administered at a dose of 1.3 mg/kg on days 1, 4, 8, 11, 29, 32, 36 and 39. When radiotherapy was completed, bortezomib was given at the same dose on days 1, 4, 8, and 11 every four weeks. The median PFS for the patients was 6.2 months, while the median OS was 19.1 months, an improvement compared with patients who received a standard STUPP treatment regimen or BEV combined with a radiotherapy-TMZ regimen (2, 6, 53). When taking MGMT promoter methylation status into account, the results differed. In patients with a methylated MGMT promoter, the median PFS was 24.7 months, and the median OS was up to 61 months, significant improvements compared with the standard protocol. The second-generation proteasome inhibitor marizomib has also been studied. An animal model showed that marizomib had better blood-brain barrier permeability and obviously prolonged the survival time of animals with glioma (54). Moreover, an ongoing multicenter, randomized phase I trial (NCT02903069) involving 72 patients with newly diagnosed GBM is being conducted to evaluate the safety and maximum tolerated dose of tumor treatment field (TTF) plus proteasome inhibitors. These results may provide new perspectives on the application of proteasome inhibitors.

From the results of this phase II trial, bortezomib seems to be a candidate for treating GBM patients, especially patients with a methylated MGMT promoter, but the results of the clinical trials are still controversial. First, bortezomib caused serious AEs: 54% of the patients developed grade 3-4 drug-related AEs, which, like regorafenib above, may cause potential risks for further expanding the scale of research. Because this drug has a sensitization effect for radiotherapy, nGBM is the most ideal research object in order to maximize the survival benefit of patients. From the perspective of trial design, this trial was also single-arm and thus had shortcomings similar to those of the clinical trial of iniparib; the control group was patients treated by the STUPP regimen and BEV combined with TMZ and radiotherapy. Moreover, all of the patients enrolled in this trial had undergone intracranial tumor resection, whereas the patients in the historical control group had not. In addition, only 24 patients were included in this study, and the sample size was significantly smaller than the historical control group, which will also affect the accuracy of statistical analysis. Overall, despite the encouraging results of this clinical trial, there are still many deficiencies. Because the drug had a significantly better therapeutic effect in the subgroup of patients with a methylated MGMT promoter, molecular markers could be used as one of the inclusion criteria for patients in future phase III clinical trials.

### BRAF Inhibitors

The BRAF gene is a member of the RAF family (ARAF, BRAF, and CRAF) and is a serine/threonine kinase. BRAF is an oncogene, and its protein product, the B-raf protein, participates in the RAS-RAF-MEK-ERK intracellular signaling pathway, which plays an important role in cell proliferation, differentiation, apoptosis and survival. BRAF mutations are associated with 15% of human cancers, including malignant melanoma, colorectal cancer, marginal ovarian cancer, etc (55). The most common gene mutation in central nervous system tumors is the BRAFV600E mutation, which can lead to a change in amino acid 600 f in the B-raf protein from glutamic acid to valine. This change greatly increases the activity of the kinase and leads to cell transformation of cells (56). The probability of this mutation in nervous system tumors is 38%-100% in pleomorphic xanthoastrocytoma (PXA), 50% in anaplastic gangliogliomas, 18%-57% in gangliogliomas, and <3% in high-grade gliomas, including GBM (57–59). In summary, inhibiting the activity of the BRAFV600E mutation can prevent the occurrence and progression of tumors to a certain extent, and vemurafenib is an inhibitor of the V600E mutation.

Thomas Kaley et al. conducted a multicenter, phase II clinical trial of vemurafenib (31). This study included 24 glioma patients with BRAFV600E mutations, including GBM, anaplastic astrocytoma, PXA, anaplastic gangliogliomas and unclassified gliomas. In this study, vemurafenib was given at 960 mg twice a day for 28 days, until the tumor progressed or until patients had intolerable side effects. The results showed that the total objective response rate was 25%, the median PFS was 5.5 months, and the median OS was 28.2 months. For the GBM and anaplastic astrocytoma cohort, PFS and OS were 5.3 months and 11.9 months, respectively, while the OS was significantly longer in the other cohorts. Among the 6 patients with GBM and 5 patients with anaplastic astrocytoma, 1 patient had partial remission, 5 patients had stable disease, and 2 patients maintained a stable disease state for more than a year. At present, the mechanism of the significant differences in the therapeutic effect of this drug in different histologic types is still being studied, but it seems like that vemurafenib has clinically meaningful activity in glioma patients with BRAFV600E mutations according to this trial.

With the increasing importance of molecular biomarkers, new types of clinical trials have been proposed by researchers, including “basket trials”. This research highlights the importance of molecular biomarkers from the perspective of design, which is very farsighted, but this design scheme also brings some disadvantages. The clinical trial of vemurafenib included glioma patients of all grades with the BRAFV600E mutation, but the total number of patients was only 24, among whom only 6 patients had GBM, which was too small. In addition, this trial was a single-arm study, so the disadvantages of using previous research data as a historical control mentioned in the discussions of the iniparib and bortezomib trials emerge. Finally, the study paid attention to BRAFV600E and ignored many other important molecular markers for glioma. Therefore, whether the conclusions of this trial are reliable remains unclear, which also serves as a wake-up call for future basket trials.

In addition, since BRAF inhibitor alone can cause tumor resistance (60, 61), the combination of BRAF inhibitor with inhibitor targeting MEK, a downstream of BRAF, may be a promising treatment regimen that can avoid the development of drug resistance (62). Currently, several clinical trials of this combination regimen have been carried out. And the interim analysis of one of them using Dabrafenib plus Trametinib in adult high-grade glioma patients shows that in WHO grade III glioma, the response rate is 22% and in GBM was 29% (63). The final conclusion of this trial may be promising.

### Mesenchymal Epithelial Transition Factor Pathway Inhibitors

MET factors are members of the receptor tyrosine kinase family, and their natural ligand is hepatocyte growth factor (HGF); together these proteins form the HGF/MET signaling pathway. The combination of MET and HGF is necessary to maintain normal physiological function and is closely regulated by the human body (64). However, this pathway is dysregulated in tumors. Researchers have found that the HGF/MET signaling pathway is abnormally activated in many malignant tumors and that this abnormal activation promotes tumor proliferation, survival and metastasis (65). According to the results of the current study, MET not only has a carcinogenic effect but can also be a predictive factor for the prognosis of patients with tumors. The growth of tumors can be blocked by inhibiting the activation of the HGF receptor and its downstream signaling pathway (66). Therefore, the MET signaling pathway is considered a potential therapeutic target, and inhibition of this pathway may be used for the targeted treatment of cancer.

Onartuzumab (Ona) is a humanized monoclonal anti-MET pathway drug, and Timothy Cloughesy et al. published a multicenter randomized double-blind phase II clinical trial on this drug in 2016 (32). This study included 129 patients with initial GBM recurrence who had not yet been treated with BEV. Researchers compared the efficacy of Ona (15 mg/kg, once every 3 weeks) plus BEV (15 mg/kg, once every 3 weeks) with that of BEV alone. Median PFS was 3.9 months in the Ona plus BEV group and 2.9 months in the BEV group, while OS was 8.8 months and 12.6 months, respectively. Among all the patients, adverse effects ≥ grade 3 were reported in 38.5% of patients in the Ona plus BEV group and in 35.9% of patients in the BEV group. Based on these results, the researchers concluded that in unscreened patients with rGBM, combining Ona with BEV does not have a significant benefit. However, the results of molecular analyses found that patients with high expression of HGF or an unmethylated MGMT promoter were more likely to benefit from the combination regimen, and their OS and PFS were significantly prolonged compared to patients with these characteristics who were treated with BEV alone.

For Ona, the most important problem is drug-related AEs; 2 patients had grade 5 AEs when treated with Ona. In recent years, in addition to GBM, clinical trials have also been conducted in non-small-cell lung cancer and Her2-negative breast cancer (67, 68). Non-small-cell lung cancer treatment with Ona completed phase III clinical trials but failed. Research on these two solid tumors found many AEs caused by this drug, similar to the results in patients with GBM. This phenomenon indicates that the AEs are not closely related to the diseases but to the drug itself, and a good solution has not yet been found. Therefore, phase III studies of the drug may need to focus more on addressing the side effects of the drug and actively looking for possible causes of severe side effects during symptomatic treatment.

The second problem with this drug study is related to patients’ response to the drug. The researchers found that in unscreened patients, the experimental group did not achieve a more significant survival benefit compared with the control group. However, when the researchers grouped the patients according to biomarkers, the advantage of the drug was clearly highlighted. This phenomenon further illustrates the value of molecular biomarkers as inclusion criteria for patients, which warrants attention to in future trials.

### CD95L Inhibitors

The CD95 receptor, also known as Fas or apoptosis antigen-1, is a member of the tumor necrosis factor (TNF) receptor superfamily. Its corresponding ligand, CD95L, is closely related to immune homeostasis and immune surveillance. Mutation of the CD95 receptor or CD95L can lead to autoimmune diseases such as systemic lupus erythematosus (SLE) and cancer (69). CD95 and CD95L are believed to play a role in immune homeostasis and tumor elimination through the apoptosis signaling pathway. However, recent studies have shown that CD95 can also stimulate nonapoptotic signaling, promote inflammation and contribute to carcinogenesis (69). Recently, researchers have observed that soluble CD95L is overexpressed in the serum of patients with triple-negative breast cancer or SLE, which affects the severity of these diseases by activating nonapoptotic signaling pathways and promoting the metastasis or accumulation of certain T cell subsets in damaged organs (70–72). Therefore, CD95L inhibitors can restrain the invasive growth of tumors by blocking CD95L on the cell membrane, and they can also regulate the number of antitumor T cells, thus inhibiting tumor growth. From another point of view, the expression of CD95L may predict the poor patient prognosis.

APG101 is a fusion protein drug that can bind to CD95L. This molecule blocks CD95L and can inhibit the effects of the binding of CD95 and CD95L. Wolfgang Wick et al. conducted a randomized phase II clinical trial of APG101 that included 91 patients with GBM (33). All patients had primary or secondary relapse. The patients were randomly divided into two groups. Patients in group one were treated with secondary radiotherapy, while patients in group two were treated with secondary radiotherapy plus APG101. The results showed that the 6-month PFS rates were 3.8% and 20.7%, respectively, suggesting that radiotherapy plus APG101 is effective for rGBM. Through further molecular analysis, the researchers found that patients with low CpG2 methylation levels at the CD95L promoter had a lower therapeutic effect than patients with high CpG2 methylation levels at the CD95L promoter. These results suggest that the methylation level of CD95L is a potential biomarker for this drug, but further research is needed to verify this conclusion.

### G Protein-Coupled Receptor Antagonists

G protein-coupled receptors are the largest receptor family on the surface of human cell membranes. These receptors regulate the physiological functions of the body by binding to extracellular ligands and regulating intracellular signal transduction. Researchers have observed dysfunction of these receptors in many diseases, so drugs targeting these receptors are valuable (73, 74).

ONC 201 is a drug targeting this receptor family that can selectively antagonize the G protein-coupled receptor DRD2 and induce p53-independent apoptosis by activating stress reactions and inactivating Akt/ERK (75, 76). ONC 201 has been confirmed to have anti-proliferation and apoptosis-promoting effects in a variety of tumor cells and does not have an obvious apoptosis-promoting effect in normal cells (77). As a result, the drug has undergone a phase II clinical trial. Isabel Arrillaga-Romany et al. conducted a phase II clinical trial in rGBM patients who had not yet been treated with BEV (34). A total of 17 patients were included in this study and were treated with ONC 201 at a dose of 625 mg every 3 weeks. The mOS was 41.6 weeks, the 6-month OS rate was 71%, the 9-month OS rate was 53%, and the 6-month PFS rate was 11.8%. As there is no standard treatment regimen for rGBM, this trial used survival data extracted from a meta-analysis, which showed that ONC 201 was well-tolerated and exerted its therapeutic effect when used alone in the treatment of rGBM patients, but this trial still leaves much to be desired (78–80).

### Stromal Cell-Derived Factor-1 Chemokine Receptor Type-4 (CXCR-4) Inhibitor

Recently, researchers have proposed a new hypothesis about the mechanism of recurrence of GBM after radiotherapy: the local hypoxia induced by radiation increases the secretion of stromal cell-derived factor-1 (SDF-1/CXCL12). This factor then binds to its chemokine receptors CXCR4 and CXCR7, causing monocytes from bone marrow to enter the tumor, thus producing new blood vessels in the tumor and leading to recurrence (81). This hypothesis was verified by further preclinical studies (82, 83). The results showed that the increase in monocyte/macrophage entry into tumor tissues that had received radiotherapy was mediated by the SDF-1/CXCR4-CXCR7 pathway, and thus inhibition of this pathway could increase the local control rate of tumors after radiotherapy and prolong the survival time of patients. This regimen was named Macrophage Exclusion after Radiation Therapy (MERT).

Based on the mechanism mentioned above, Reena P. Thomas et al. conducted a phase I/II clinical trial in patients with newly diagnosed GBM using the CXCR-4 inhibitor plerixafor (35). A total of 29 patients were included in this trial. The administration of plerixafor began on the 35th day of concurrent chemoradiotherapy and was infused intravenously through a PICC for 4 weeks at a dose of 400 μg/kg/d. The results of the clinical trial showed that plerixafor was well-tolerated, and no serious AEs were found in any of the 29 patients. Regarding survival time, the PFS and OS were both longer in these patients than in the historical control group treated with the STUPP regimen. The median PFS of these patients was 14.5 months, and the median OS was 21.3 months. This study seems encouraging and may become the foundation for future phase III clinical trials.

The last three clinical trials are briefly analyzed here. Firstly, the clinical trials of ONC201 and plerixafor were single-arm trials and thus have the corresponding disadvantages. In addition, only 17 patients were included in the ONC 201 study; this small sample size is another shortcoming. The clinical trial for APG101 did not further analyze the molecular pathology results, which should be further improved in future trial design.

## Discussion and Perspectives

Although many phase II and phase III clinical trials of molecular-targeted drugs have been completed in GBM patients, all have some drawbacks. For completed phase III clinical trials, these drawbacks may be directly related to their failure to obtain positive results, while for phase II clinical trials, these drawbacks may pose a significant obstacle to further research. In this section, we will summarize the deficiencies mentioned above in order to promote future research.

The cause of phase III clinical trial failure has always been one of the issues of greatest concern for clinicians and researchers, and our review of the literature reveals that the cause of failure can be divided into two aspects: failure caused by the mechanism of the drug itself and defects of the trial design. We take the most widely studied VEGF inhibitor, BEV, as an example to illustrate the first point. Some angiogenesis pathways in the tumor microenvironment, such as vessel cooperation, vascular mimicry, and vascular intussusception, cannot be blocked by VEGF inhibitors (84). In addition, BEV not only has anti-tumor effects but also acts on some pro-tumor pathways. For example, VEGF can inhibit the c-MET pathway; thus, anti-VEGF drugs may block this pathway and promote tumor development. Moreover, researchers have found that BEV can increase the level of MMP and thus promote the tumor’s aggressiveness (85). Therefore, further research may need to focus on the original characteristics of tumors and drugs. Combined application of immunotherapy or other targeted drugs may be a good future research direction.

EGFR inhibitors, which have also been extensively studied in GBM, also present this kind of problem. As mentioned above, nimotuzumab is the only drug that has entered phase III trials but still failed. First, TKIs are not that specific and often act on several tyrosine kinases, albeit with differential efficacy. The EGFR target itself also has weak antigenicity, which limits its ability to cause a long-lasting and stable effect; this ability declines even further with tumor development (86). Moreover, during the treatment process, the long-term use of EGFR inhibitors will up-regulate the expression of downstream PDGFRβ, thus activating the urokinase receptor-Bim signaling pathway and the TNF-JNK-Axl-ERK signaling pathway (87). Multiple mechanisms can trigger tumor resistance to EGFR-targeted therapy. Enhancing the antigenicity of EGFR targets and the specificity and stability of targeted drugs are problems warranting attention in the future.

In addition, due to the extensive tumor heterogeneity in GBM, current drugs targeting only one specific target may not be effective (88). However, current studies of combination targeted therapy have shown that the toxicity is greater than monotherapy, and it also has higher requirements for the detection of molecular markers (89, 90). Therefore, how to solve the above problems in the future, as well as the development of more safe and effective multi-target inhibitors will be the research direction of targeted drugs.

The failure of the phase III clinical trial was also due to the deficiencies in trial design. First of all, it should be mentioned that there is no clear standard for the suitability of targeted drugs to enter phase III clinical trials in GBM patients. Consequently, there are serious problems in the selection of drugs, which are mainly reflected in the following four aspects (91): 1) conducting a phase III clinical trial without completing a phase II trial; 2) conducting a phase III clinical trial based on negative results of a phase II trial; 3) failing to obtain a positive result in a phase II clinical trial for recurrent disease but conducting a phase III clinical trial for newly diagnosed disease; 4) obtaining a benefit in a subgroup of patients in a phase II clinical trial but applying the same regimen to all patients without selection in a subsequent phase III trial. For example, the PKC and PI3K/AKT inhibitor enzastaurin entered a phase III clinical trial before finishing phase II trials, which might be responsible for the failure of the phase III trial. The phase III trial was conducted from March 2006 to August 2007 based on the encouraging interim data which showed a relatively high objective radiographic response rate of 20% (14), however, the result of the phase II trial released in 2015 suggesting PFS of enzastaurin in combination with BEV did not surpass BEV monotherapy (92). Second, patients outcomes in clinical trials are often evaluated by RANO currently (93), but these criteria do not always seem to be reliable, so there may be pseudo-response or pseudo-progression. As discussed above, BEV plays an anti-tumor role mainly through anti-angiogenesis, but this effect may confuse clinicians when assessing the response to treatment by MRI, leading to a great impact on the evaluation of therapeutic effect. After treatment, 20%-30% of GBM patients will show pseudo-progression on imaging (93), which further increases the difficulty of imaging evaluation of disease. Imaging indicators that can reduce misunderstanding among clinical workers should be considered in future trial design, especially for drugs with anti-angiogenesis effects (94–96).

The above analysis of the reasons for failure of phase III clinical trials clearly indicates that many of these problems are closely related to phase II clinical trials. Therefore, better design of phase II clinical trials is a prerequisite for the success of phase III trials. We have already analyzed the defects of some phase II clinical trial with positive results above, so in this section, we will summarize these defects.

In terms of the drugs themselves, the most important issue in phase II trials is drug-related AEs. As mentioned above, in the phase II clinical trial of Ona, 25 patients (38.5%) developed grade 3-4 drug-related AEs, and 2 (3.1%) even developed grade 5 AEs. The drug also showed severe AEs during the treatment of other cancers (67, 68). The above phenomenon indicates that side effects of this drug are a common problem and have not been solved effectively. Similar problems were observed for regorafenib and bortezomib (28, 30), which caused grade 3-4 AEs in 56% and 54% of patients, respectively, and an effective solution has not yet been found. If this problem is not properly solved, future studies may be severely limited.

From the perspective of trial design, there are two main problems in current phase II clinical trials. The first serious problem is the frequent use of a “single-arm” design. Among the 8 phase II clinical trial with positive results, five were single-arm trials. As mentioned above, this kind of trial faces the serious problem of choosing historical control groups. The disadvantages of using historical control groups include the following: 1) with the continuous improvement of comprehensive treatment and medical care, longer survival than historical controls is not necessarily the result of the drug being studied. The survival data of the phase III EORTC study is always used as the historical comparison in single arm phase II trials, however EORTC was conducted in Europe and Canada when experience of temozolomide was limited and standard care of GBM patients was lacking, which may account for the poorer results compering to later phase II trials (47).; 2) the calculation of OS may differ between the experimental groups and the historical control groups. In the phase II trials of iniparib with concurrent chemoradiation in patients with newly diagnosed GBM, the survival was calculated from the date of diagnosis (29); however, randomized trials, which were used as historical control groups, calculated survival from the date of randomization (2, 17, 46), leading to at least 1-month diversity in mOS calculation.; 3) rGBM patients in the experimental group and patients in the historical control group may have had different treatment plans in the early period, leading to unreliable comparisons. In the phase II trial of bortezomib in combination with temozolomide and regional therapy of patients with newly-diagnosed GBM (30), the enrolled patients all underwent surgical resections, which was not consistent with most of the historical studies. In stupp’s study, 17% of patients had only biopsy without surgical resections (2)and this difference might contribute to prolonged survival data in the phase II trial.; 4) the sample sizes in phase II studies are small, while the historical control data are often from phase III clinical trials with large sample sizes, which may cause statistical inaccuracy when comparing. Only 17 patients were enrolled in the phase II study of ONC201 (34) and the researchers utilized meta-analyses as historical controls with 1348, 596 and 338 enrolled patients respectively (78–80). The huge diversity would cause unreliable results. 5) molecular diagnosis of glioma was introduced into WHO classification in 2016, therefore, detailed molecular pathological data are often not available in the historical control group, such as *MGMT* promoter methylation, *IDH* mutation, or *CDKN2A/B* deletion status (31).In view of the disadvantages of the above-mentioned single-arm trials, the currently recommended trial design scheme for clinical trials is a randomized trial (97).

The second problem relates to the use of molecular biomarkers in the trial design. The introduction of the WHO classification in 2016 elevated molecular markers. Consequently, many new types of clinical trials based on molecular biomarkers have gradually been carried out, such as the clinical trial of the BRAFV600E mutation inhibitor vemurafenib mentioned above, which was a “basket trial”. However, the trial was still single-arm, and the number of patients included was very limited. The question that emerges from this study is that the use of molecular biomarkers as inclusion criteria may require larger study sample sizes and better control group selection in the future. However, unlike this basket trial, most current studies have not included molecular biomarkers as inclusion criteria but only conducted post-hoc analysis. In a retrospective analysis, researchers found that in a subgroup of patients classified by molecular biomarkers, some drugs produced significantly better therapeutic effects than other subgroups, such as Ona in rGBM patients with high HGF expression and an unmethylated MGMT promoter. ONC201 may lead to a stronger therapeutic response in patients with a H3K27M mutation, and a literature review on this issue has been published recently (98).

Of course, this research strategy for finding biomarkers through post-hoc analysis also has a certain development prospect after the application of new clinical trial statistical methods, such as the Individualized Screening Trial of Innovative Glioblastoma Therapy (INSIGhT) trial and the GBM Adaptive, Global, Innovative Learning Environment (AGILE) consortium (99). The significance of molecular markers is self-evident whether they are the inclusion criteria of clinical trials or the important factor of post-hoc analysis, and they are bound to occupy an irreplaceable position in future clinical trials.

In addition to the above two main points, the current phase II clinical trials of targeted drugs for GBM could also be improved by selecting more appropriate endpoints and adjusting the evaluation criteria for study results. As imaging indicators are still predictive and are changing with the rise of immunotherapy, it is not appropriate to use them as an endpoint of the study. Besides, given the high failure rate of phase III clinical trials, a higher threshold of success may enable researchers to better control the criteria for entry of drugs into phase III trials (100).

In recent years, the failure of clinical trials of molecular targeted drugs has promoted the exploration of the intrinsic mechanisms of these drugs. At the same time, immunotherapy and TTF therapy are on the rise, so more and more researchers are focusing on the combination of molecular targeted drugs with immunotherapy or TTF. Ongoing clinical trials are giving special consideration to the use of EGFR as an immunologic target. The ReACT trial of rindopepimut is one examples. Researchers remain steadfast believers in the power of immunotherapy to transform EGFR treatment. Moreover, TTF are now FDA-approved for high-grade glioma treatment. A novel application of this treatment modality is being assessed for GBMs. Clinical phase II trials are being conducted to test the efficacy of this treatment modality as adjuvant therapy to nivolumab for rGBM not treated with BEV (NCT03430791) and to BEV for BEV-refractory rGBM (NCT02663271, NCT02743078, NCT01894061). The advent of combined therapies may revolutionize GBM treatment.

Based on the completed or ongoing phase II clinical trials mentioned above and summarized in **Table 2**, we found that patients may benefit more from certain targeted drugs after molecular subtyping, further confirming the importance of molecular detection in the diagnosis of glioma patients in the future. At present, many promising targeted drugs are being studied in phase III clinical trials. However, further improvements in trial design, larger sample sizes, and a deeper understanding of molecular subtyping are still needed.

## Author Contributions

All authors designed and conducted this review. YW and WM had primary responsibility for the final content. YNW, WC and YS wrote the original draft. WM and YW supervised the writing of the manuscript. CY, ZK and YKW assisted on critical revision of the article for important intellectual content. YNW, WC and YS equally share the first authorship. WM and YW equally share the corresponding authorship. All authors contributed to the article and approved the submitted version.

## Funding

This research was funded by The Chinese Academy of Medical Sciences Innovation Fund for Medical Sciences [grant number: 2016-I2M-2-001].

## Conflict of Interest

The authors declare that the research was conducted in the absence of any commercial or financial relationships that could be construed as a potential conflict of interest.

## Publisher’s Note

All claims expressed in this article are solely those of the authors and do not necessarily represent those of their affiliated organizations, or those of the publisher, the editors and the reviewers. Any product that may be evaluated in this article, or claim that may be made by its manufacturer, is not guaranteed or endorsed by the publisher.
